# Novel surgical approach based on the sentinel node concept in patients with early gastric cancer

**DOI:** 10.1002/ags3.12027

**Published:** 2017-08-31

**Authors:** Shoji Natsugoe, Takaaki Arigami, Yoshikazu Uenosono, Shigehiro Yanagita

**Affiliations:** ^1^ Department of Digestive Surgery, Breast and Thyroid Surgery Field of Oncology Kagoshima University Graduate School of Medical and Dental Sciences Kagoshima Japan; ^2^ Molecular Frontier Surgery Course of Advanced Therapeutics Kagoshima University Graduate School of Medical and Dental Sciences Kagoshima Japan

**Keywords:** fluorescence imaging, gastric cancer, laparoscopic and endoscopic cooperative surgery, lymph node metastasis, sentinel node navigation surgery

## Abstract

Recent prospective multicenter trials have demonstrated the clinical safety and efficacy of sentinel node navigation surgery (SNNS) in patients with early gastric cancer. Further, development of an intraoperative imaging system and an indocyanine green fluorescence imaging approach has been attracting attention as a novel tool for detection of the sentinel node (SN). The greatest advantage of an in vivo imaging system is that it visualizes SN and afferent lymphatic vessels from the primary tumor site more clearly than the conventional dye approach. Besides visualization of the SN, it is also essential to accurately assess the presence or absence of lymph node metastasis in the intraoperative management of SNNS. However, the clinical significance of lymph node micrometastasis (LNM) in patients with gastric cancer remains controversial. Reverse transcription‐polymerase chain reaction (RT‐PCR) is one of the representative assays used to identify LNM. A rapid RT‐PCR assay that completes the detection of LNM within approximately 40 minutes has recently been produced and applied in the clinical management of SNNS. From the viewpoint of surgical methods, modified laparoscopic and endoscopic cooperative surgery with non‐exposed approaches has recently been highlighted as a promising technique to prevent tumor dissemination caused by surgical procedures, and is likely to be clinically applied to SNNS in the future. When carrying out SNNS as a minimally invasive surgery, it is important to consider the balance between post‐surgical quality of life and curability. Future prospective studies on SNNS will greatly contribute to furthering its establishment as a beneficial procedure for patients with early gastric cancer.

## 1 INTRODUCTION

Gastric cancer is one of the most common malignancies in Japan.^1^ Incidence of patients with early gastric cancer has increased as a result of recent advances in endoscopic diagnostics using narrow‐band imaging.^2^ Indications for endoscopic submucosal dissection (ESD) are based on the criteria of the Japanese Gastric Cancer Treatment Guidelines 2014 (ver. 4), and extended indications for ESD in patients with early gastric tumors without lymph node metastasis have been clinically discussed.^3^, ^4^ According to these guidelines, standard gastrectomy with lymphadenectomy is still recommended for patients with tumors and no indications for ESD.^3^ In clinical practice, therapeutic strategies need to be designed in order to prevent lymph node recurrence after ESD.

Surgical gastrectomy with lymphadenectomy is currently regarded as a standard treatment for gastric cancer, even for some patients with early gastric cancer. However, conventional surgical treatments are associated with the development of post‐gastrectomy syndrome in some patients. Therefore, it is important to consider the balance between post‐surgical quality of life (QOL) and curability when selecting surgical procedures for the clinical management of patients with early gastric cancer. A large number of surgeons have recently focused on function‐preserving gastrectomy as a promising procedure that considers QOL. For example, pylorus‐preserving gastrectomy, segmental gastrectomy, and local resection are representative examples of function‐preserving gastrectomy. According to the Japanese Gastric Cancer Treatment Guidelines 2014 (ver. 4), these procedures are defined based on the stomach volume to be resected.^3^ However, when planning function‐preserving gastrectomy, limited lymphadenectomy is a key concern from the viewpoint of disease curability. Moreover, the accuracy of detecting lymph node metastasis using preoperative examinations is clinically inadequate in patients with gastric cancer.^5^

The sentinel node (SN) concept was initially proposed by Morton et al.^6^ for patients with melanoma. SN are defined as the first lymph nodes to receive lymphatic flow from primary tumor sites. Many investigators have already demonstrated the clinical utility of sentinel node navigation surgery (SNNS) for patients with gastric cancer,^7^, ^8^, ^9^, ^10^, ^11^ and the current technical procedures for SNNS have been gradually advancing over time. Furthermore, intraoperative methods have been developed for the accurate diagnosis of lymph node metastasis, including micrometastasis.^12^ This review will focus on a novel technique for and advances in SNNS in patients with early gastric cancer.

## 2 PROSPECTIVE MULTICENTER TRIALS ON SNNS

Many researchers have reported retrospective findings on SNNS for patients with gastric cancer.^8^, ^9^, ^10^ However, limited information is available on the accuracy of the SN concept based on prospective multicenter trials.

Table 1 summarizes three prospective multicenter studies reported since 2013 on SNNS for patients with gastric cancer.^13^, ^14^, ^15^ A prospective multicenter trial by the Japan Society of Sentinel Node Navigation Surgery assessed the clinical safety and efficacy of SNNS using the endoscopic dual tracer method in 397 patients with previously untreated cT1 or cT2 gastric cancer measuring <4.0 cm in diameter.^13^ In this prospective study, SN detection and accuracy rates were 97.5% (387/397) and 99.0% (383/387), respectively.^13^ Although four patients had false‐negative results in terms of SN detection, pathological examinations showed pT2 or tumors measuring >4.0 cm in three patients.^13^ Accordingly, this study group concluded that SNNS is applicable to patients who are preoperatively diagnosed with cT1 and cN0 gastric cancer measuring <4.0 cm in diameter.^13^

**Table 1:** Prospective multicenter trials on sentinel node navigation surgery for patients with gastric cancer

Year	Study	No. patients	Median age, y (range)	Sex (male/female)	Tumor location (upper/middle/lower/unknown)	cT factor	Median tumor size (mm, range)	Tracers	SN detection rate, %	Assessment of the SN concept, %			Clinical validity of SNNS
										Sensitivity	False‐negative rate	Accuracy rate	
2013	Kitagawa et al.^13^	397	63 (29‐87)	264/133	76/176/145/0	cT1‐T2	30 (6‐100)	Dual (radioisotope and dye)	97.5 (387/397)	93 (53/57)	7 (4/57)	99.0 (383/387)	Yes
2014	Miyashiro et al.^14^	440	62 (26‐80)	285/155	29/249/162/0	cT1	23 (0‐180)	Dye	97.7 (304/311)	86 (24/28)	14 (4/28)	98.7 (300/304)	‐
2015	Lee et al.^15^	108	55.3	51/57	1/46/60/1	cT1‐T2	25	Dual (radioisotope and dye)	92.6 (100/108)	100 (10/10)	0 (0/10)	100 (100/100)	Yes

SN, sentinel node; SNNS, sentinel node navigation surgery; ‐, not determined.

The Japan Clinical Oncology Group (JCOG) conducted a multicenter trial (JCOG0302) to assess the accuracy of SN biopsy in patients with cT1 gastric cancer measuring <4.0 cm at the maximum diameter.^14^ In the JCOG0302 trial, SN were identified using an intraoperative serosal injection of indocyanine green (ICG) and lymph node metastasis was intraoperatively evaluated by hematoxylin‐eosin (HE) staining using only a single section.^14^ Although 440 patients were enrolled in the study, the multicenter trial was suspended because of the high false‐negative rate (46%).^14^ However, they reported that the false‐negative rate decreased to 14% when using additional sections for pathological assessments.^14^ The main reason for this change in the rate of false‐negatives with additional analysis is likely the heterogeneous distribution of metastatic foci in lymph nodes.^12^ These findings demonstrated the clinical importance of pathological examinations using multiple sections for the intraoperative diagnosis of lymph node metastasis. Consequently, they concluded that intraoperative HE assessments using only one section were inadequate to accurately diagnose the metastatic status of SN.^14^

A Korean group conducted a prospective multicenter feasibility study as prerequisite quality control for the surgical standardization of laparoscopic sentinel node basin dissection (SBD) before starting a phase III trial (SENORITA trial).^15^ This study included 108 patients who had cT1 or cT2 tumors measuring <4.0 cm in diameter for the final analysis.^15^ In this quality control study, the SN detection rate, sensitivity, and false‐negative rate were 92.6% (100/108), 100%, and 0%, respectively.^15^ These findings indicated that SBD is a feasible tool for patients with early gastric cancer, and planned the SENORITA trial to assess oncological outcomes, postoperative morbidity and mortality, and QOL between laparoscopic stomach‐preserving surgery with SBD and laparoscopic standard gastrectomy.^15^

The clinical validity of the SN concept in patients with cT1 and cN0 gastric cancer has, thus, been adequately confirmed by clinical studies on SNNS, including retrospective analyses. As patients with early gastric cancer have a better prognosis after standard treatments, SNNS based on individualized lymphadenectomy should be safely carried out to prevent lymph node recurrence. Further standardization of clinical procedures will contribute greatly to the development of SNNS for patients with early gastric cancer.

## 3 A NOVEL IMAGING SYSTEM DEVELOPED FOR SN DETECTION

The performance of intraoperative imaging systems for SN detection has been markedly improved by recent advances in medical instruments. The ICG fluorescence imaging approach has been attracting attention in this field. The greatest advantage of the ICG fluorescence imaging system is that it allows visualization of SN and afferent lymphatic vessels from primary tumor sites more clearly than a conventional dye approach based on visual recognition without special tools. To date, several investigators have demonstrated the clinical benefit of the ICG fluorescence imaging approach for SN detection in patients with several malignancies, including gastric cancer.^16^, ^17^, ^18^, ^19^

**Figure 1:**
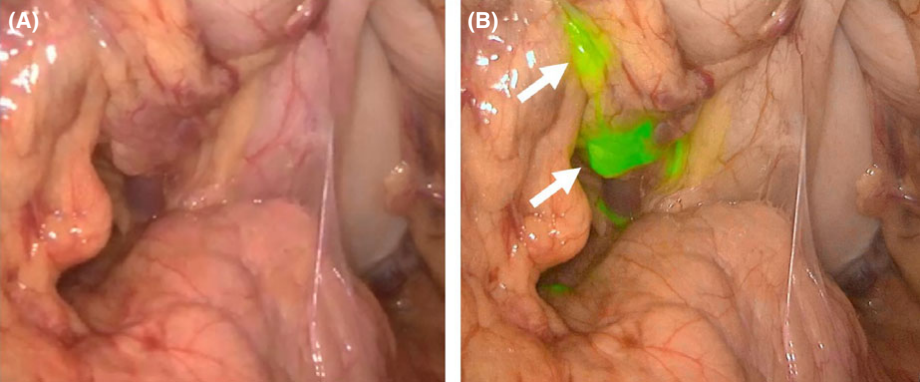
Laparoscopic indocyanine green fluorescence imaging system. (A) Conventional white light. (B) Near‐infrared fluorescence. This case had two sentinel nodes in stations no. 3 and no. 7 (arrows).

From June 2012, we introduced a laparoscopic ICG fluorescence imaging system when carrying out SNNS. The PINPOINT Endoscopic Fluorescence Imaging System (Novadaq, Mississauga, ON, Canada) is currently used for SN detection in our institution. This system allows visualization of high‐definition images under conventional white light and near‐infrared fluorescence using ICG.^20^ In our laparoscopic procedures using this system, fluorescent lymph nodes are clearly identified, even when none of the stained lymph nodes are detected under white light (Figure 1A,B). Fluorescent lymph nodes are then marked by clips, and lymphatic basin dissection, including fluorescent lymph nodes and afferent lymphatic vessels from the primary tumor, is carried out. We previously reported that SN were confirmed in all patients who underwent SNNS based on the ICG fluorescence imaging system.^21^ We believe that the ICG fluorescence imaging system is essentially a safe procedure for future use in SNNS.

## 4 INTRAOPERATIVE DIAGNOSTIC TECHNOLOGIES FOR ASSESSING SN METASTASIS

Lymph node metastasis is one of the most significant prognostic indicators in patients with gastric cancer.^22^, ^23^ Therefore, it is important to accurately assess the presence or absence of lymph node metastasis in the clinical management of patients with gastric cancer. Lymph node micrometastasis (LNM) is defined by the criteria of the tumor‐node‐metastasis classification established by the International Union Against Cancer in 2002.^24^ This criteria classifies LNM as isolated tumor cells (ITC) or micrometastasis based on the size of the metastatic tumor foci in lymph nodes.^24^ Accordingly, ITC and micrometastasis are single tumor cells or small clusters of cells measuring ≤0.2 mm and tumor cell clusters measuring from 0.2 mm to 2.0 mm in greatest dimension, respectively.^24^ The clinical impact of LNM currently remains controversial in patients with gastric cancer.^12^ Yanagita et al.^25^ investigated the proliferative activity of LNM using an immunohistochemical analysis with Ki‐67 from the viewpoint of molecular oncology. They demonstrated that the Ki‐67 positivity rate in LNM was 92%.^25^ These findings indicate that micrometastatic tumor cells within lymph nodes exhibit the ability to proliferate. Therefore, we consider a precise intraoperative diagnosis for detecting LNM by molecular methods to be required, at least when planning SNNS.

Reverse transcription‐polymerase chain reaction (RT‐PCR) is a representative tool to identify LNM. RT‐PCR assays are used for the intraoperative diagnosis of LNM in SNNS because of their ability to provide a rapid analysis. Recent advances in technology have led to the development of several RT‐PCR systems to fulfill this key requirement. One‐step nucleic acid amplification and SmartCycler (Cepheid, Sunnyvale, CA, USA) both complete the detection of LNM within approximately 40 minutes.^26^, ^27^, ^28^ Our study demonstrated that the sensitivity and specificity of the SmartCycler system are high as a result of multiplex assays using the double markers of carcinoembryonic antigen (CAE) and cytokeratin‐19.^26^ At our institution, we use a rapid RT‐PCR assay using SmartCycler as well as HE staining for the intraoperative diagnosis of SN metastatic status when carrying out SNNS.

## 5 LAPAROSCOPIC AND ENDOSCOPIC COOPERATIVE SURGERY BASED ON THE SN CONCEPT

Laparoscopic and endoscopic cooperative surgery (LECS) has been developed as a novel surgical technique for patients with gastric submucosal tumors, such as gastrointestinal stromal tumors.^29^, ^30^, ^31^ As the classical LECS procedure devised by Hiki et al.^31^ results in an open gastric lumen by intentional perforation, it is useful in patients with a low risk of intra‐abdominal tumor dissemination, such as tumors that are covered by normal gastric mucosa. However, it is clinically difficult to adapt classical LECS to patients with gastric cancer because of the risk of tumor dissemination caused by opening the stomach. In order to prevent tumor dissemination induced by surgical procedures, modified LECS has recently been focused on as a promising non‐exposed approach.^31^ Non‐exposed endoscopic wall‐inversion surgery (NEWS) and the combination of laparoscopic and endoscopic approaches to neoplasia with a non‐exposure technique (CLEAN‐NET) are some representatives of modified LECS.^32^, ^33^, ^34^

**Figure 2:**
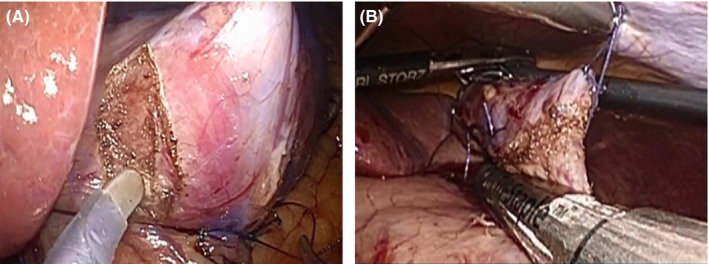
Combination of laparoscopic and endoscopic approaches to neoplasia with a non‐exposure technique (CLEAN‐NET). (A) Seromuscular dissection. (B) Full‐thickness resection

In recent years, we have carried out CLEAN‐NET with SBD for selected patients with cT1 and cN0 gastric cancer measuring <4.0 cm in diameter. According to our criteria regarding the indications for CLEAN‐NET with SBD, tumors located in the pylorus are judged as non‐indicative lesions as a result of postoperative stenosis of the gastric lumen as part of local resection. Moreover, a dual‐tracer method using ^99m^technetium‐tin colloid and ICG is basically adopted for the detection of SN. We then carry out SBD, including resection of SN identified by the laparoscopic ICG fluorescence imaging system. After confirming a pathologically negative status in SN by HE staining and RT‐PCR, CLEAN‐NET is subsequently conducted to remove primary tumors. For this, serosal markings are placed approximately 20 mm outside the lesion using a monopolar electrocautery knife under laparoscopy. In order to prevent a gap between the mucosa and muscularis propria during serosal traction, full‐thickness fixing of the gastric wall is done using 3‐0 absorbable sutures at four sites around the tumor. These stay sutures are also used for counter‐traction. The seromuscular layer is then dissected along the outside of the four stay sutures using a spatula‐shaped electrocautery knife under laparoscopy (Figure 2A). A full‐thickness specimen including the tumor is dissected using a laparoscopic stapling device (Figure 2B). The most important factor in surgical procedures involving full‐thickness resection is the establishment of a suture line from the greater to the lesser curvature in order to prevent postoperative stenosis.

In future, the indications for modified LECS with a non‐exposed approach, such as NEWS and CLEAN‐NET, will be progressively extended to patients with early gastric cancer meeting the indications for SNNS. If this method will be further stylized from the standpoint of surgical procedures, it may come to be regarded as a standard surgical tool in SNNS.

## 6 FUTURE PROSPECTS FOR SNNS AS A MINIMALLY INVASIVE APPROACH

A prospective multicenter phase II trial of personalized surgery based on a diagnosis of metastasis to SN is ongoing in Japan. This clinical study will advance medical care and, hence, we await its findings expectantly. If SNNS is clinically developed as a safe surgical approach based on the findings obtained, the clinical application of SNNS will be extensively extended among patients with early gastric cancer. Furthermore, advances have been made in endoscopic treatments for patients with early malignancies, including gastric cancer.^35^, ^36^ Therefore, ESD with laparoscopic SBD may become the ultimate stomach‐preserving surgery for avoiding lymph node recurrence in selected patients with extended indications for ESD; however, further studies are needed prior to its expansion as a surgical approach.

In conclusion, we need to assess the patients’ post‐surgical QOL in order to confirm the clinical benefits of function‐preserving gastrectomy for patients undergoing SNNS. If the benefits of SNNS on QOL are substantiated by prospective multicenter trials, minimally invasive surgery with individualized lymphadenectomy may become the recommended procedure for early gastric cancer in consideration of the balance between QOL and curability. Moreover, future studies on the surgical techniques used will greatly facilitate the establishment of SNNS for patients with early gastric cancer.

## DISCLOSURE

Conflict of Interest: Authors declare no conflicts of interest for this article.
